# Cell-extracellular matrix interactions regulate neural differentiation of human embryonic stem cells

**DOI:** 10.1186/1471-213X-8-90

**Published:** 2008-09-22

**Authors:** Wu Ma, Tara Tavakoli, Eric Derby, Yevgeniya Serebryakova, Mahendra S Rao, Mark P Mattson

**Affiliations:** 1 Stem Cell Center, Developmental Biology, American Type Culture Collection (ATCC), Manassas, VA, USA; 2 Stem Cells and Regenerative Medicine (Research), Invitrogen, Carlsbad, CA, USA; 3 Laboratory of Neurosciences, National Institute on Aging Intramural Research Program, Baltimore, MD, USA

## Abstract

**Background:**

Interactions of cells with the extracellular matrix (ECM) are critical for the establishment and maintenance of stem cell self-renewal and differentiation. However, the ECM is a complex mixture of matrix molecules; little is known about the role of ECM components in human embryonic stem cell (hESC) differentiation into neural progenitors and neurons.

**Results:**

A reproducible protocol was used to generate highly homogenous neural progenitors or a mixed population of neural progenitors and neurons from hESCs. This defined adherent culture system allowed us to examine the effect of ECM molecules on neural differentiation of hESCs. hESC-derived differentiating embryoid bodies were plated on Poly-D-Lysine (PDL), PDL/fibronectin, PDL/laminin, type I collagen and Matrigel, and cultured in neural differentiation medium. We found that the five substrates instructed neural progenitors followed by neuronal differentiation to differing degrees. Glia did not appear until 4 weeks later. Neural progenitor and neuronal generation and neurite outgrowth were significantly greater on laminin and laminin-rich Matrigel substrates than on other 3 substrates. Laminin stimulated hESC-derived neural progenitor expansion and neurite outgrowth in a dose-dependent manner. The laminin-induced neural progenitor expansion was partially blocked by the antibody against integrin α6 or β1 subunit.

**Conclusion:**

We defined laminin as a key ECM molecule to enhance neural progenitor generation, expansion and differentiation into neurons from hESCs. The cell-laminin interactions involve α6β1 integrin receptors implicating a possible role of laminin/α6β1 integrin signaling in directed neural differentiation of hESCs. Since laminin acts in concert with other ECM molecules *in vivo*, evaluating cellular responses to the composition of the ECM is essential to clarify further the role of cell-matrix interactions in neural derivation of hESCs.

## Background

Increasing evidence has shown that stem cell development requires a niche – a local microenvironment housing stem cells that regulates their self-renewal and fate in developing tissues or organs [1-5]. The regulatory signals from a niche are provided by niche cells, soluble factors and the extracellular matrix (ECM). Despite many studies showing that soluble factors such as FGFs, BMPs and Wnts can regulate stem cell behavior, the role of cell-matrix interactions in stem cell development is poorly understood. The ECM as a major niche element provides not only a scaffold for cellular support, but also an immediate microenvironment that triggers regulatory signals to support stem cell proliferation, migration and fate decision [6-8]. The ECM is a complex mixture of matrix molecules which are typically large glycoproteins, including the fibronectins, collagens, laminins and proteoglycans that assemble into fibrils or other complex macromolecular arrays. Cell adhesion to the ECM transmits extracellular signals to stem cells via integrin receptors which are heterodimeric receptors generated by selective pairing between 18 α and 8 β subunits. The complexity of the extracellular environment is revealed by examination of the special and temporal expression of patterns of ECM components and some of their cell surface receptors in the developing central nervous system (CNS) and the peripheral nervous system. Abundant ECM is present at the time when neural progenitors differentiate, migrate and neuronal axons elongate, but expression of ECM proteins is substantially reduced by the end of development [9,10]. The diversity of cell interactions with complex ECM components in the developing CNS challenges us to understand the role of cell-matrix interactions in neural differentiation of stem cells.

The ability of embryonic stem (ES) cells to generate neural cell types *in vitro *offers a powerful tool to study how the cell-ECM interactions regulate neural stem cell specification and lineage choice. Recent studies on mouse embryonic stem cells (mESCs) showed that ECM signaling influences the developmental fate of pluripotent stem cells, and the temporally restricted cell-ECM interactions direct fate and specification of neural precursors derived from mESCs [11,12]. In the present study, we used a reproducible, chemically-defined adherent culture system to direct highly purified neural commitment from human embryonic stem cells (hESCs). The robust neuroectodermal cells in neural rosettes were generated and further differentiated into neural progenitors and neurons; glial cells did not appear until 4 weeks later. This system allowed us to study quantitatively how ECM components affect the neural progenitor generation and migration from hESCs and the neurite outgrowth of developing neurons. Among the 5 substrates tested (poly-D-lysine, fibronectin, laminin, collagen and Matrigel) hESC-derived neural progenitor expansion, migration and differentiation into neurons were significantly greater on laminin and laminin-rich Matrigel than on other substrates. Laminin stimulated hESC-derived neural progenitor expansion, neuronal generation and neurite outgrowth in a dose-dependent manner. The laminin-induced neural progenitor expansion was partially blocked by antibody against integrin α6 or β1 suggesting that laminin/α6β1integrin signaling plays a critical role in the directed neural differentiation of hESCs.

## Results

### Derivation of highly homogenous neural progenitors and neurons from hES cells

**Figure 1:**
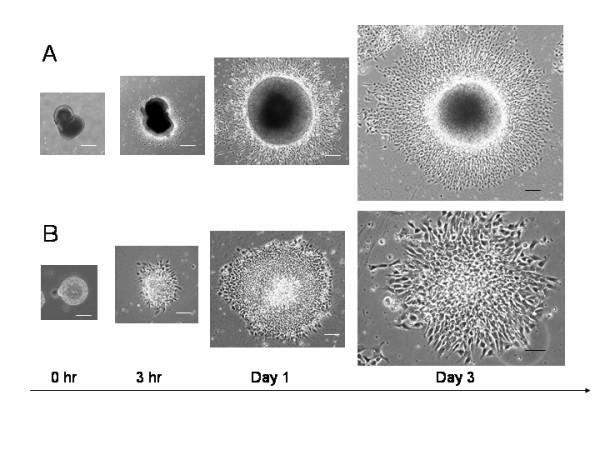
**Time-lapse series of two distinct patterns of hESC-derived neural differentiation on PDL/Laminin substrates**. Phase contrast images of cell expansion at 0 hr, 3 hr, 1 day and 3 days postplating. Some hES cell-derived embryoid bodies (EBs) at 15 days of differentiation show a solid, dark core (dark EBs) (A at 0 hr postplating) while many EBs at 17–20 days of differentiation exhibit light, transparent appearance (light EBs) (B at 0 hr postplating). (A) From a dark EB, new cells constantly generate and radially migrate away from the center of the EB and form a rim of cells. These expanded cells are mixed neural progenitors and neurons (see Fig. 2). (B) light EB gives rise to a homogenous cell population composed of almost all neural progenitors (See Fig. 3). Scale bars: in (A) = 200 μm; in (B) = 100 μm.

**Figure 2:**
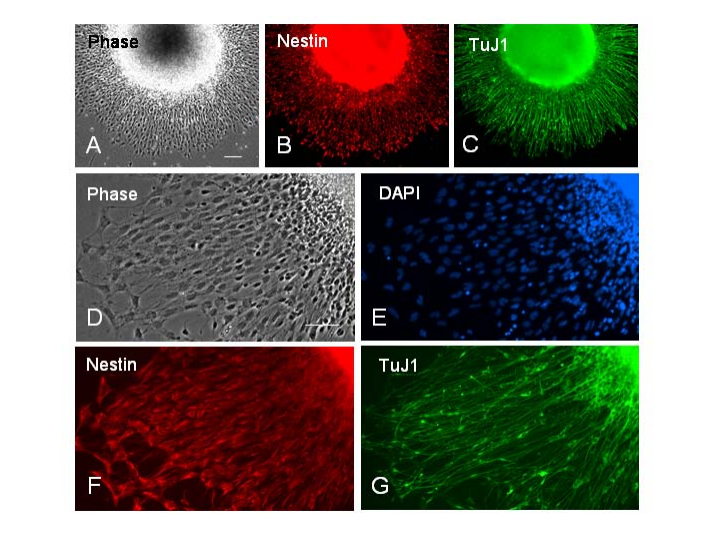
**A mixed population of neural progenitors and neurons derived from a dark EB**. The dark EB derived from TE03 cell line was plated on PDL/laminin and cultured in the neural differentiation medium (NDM) for 3 days. (A-C) Double-immunostaining of a hESC-defferentiated aggregate for nestin and TuJ1. Newly generated nestin+ and TuJ1+ cells are migrated radially away from the center of aggregates, forming a rim of neural cells around the EB sphere. (D-G) Higher magnification of differentiated cells in (A) showing a large number of nestin+ cells (F) and a few TuJ1+ neurons (G). To estimate the percentage of differentiated cells expressing nestin or TuJ1, the number of labeled cells was counted from a double-immunolabeled culture and normalized with the total number of cells determined by counting DAPI nuclear counterstained cells (E) that overlap phase-dark (D) cells. Scale bars: in (A-C) = 200 μm; in (D-G) 100 μm.

**Figure 3:**
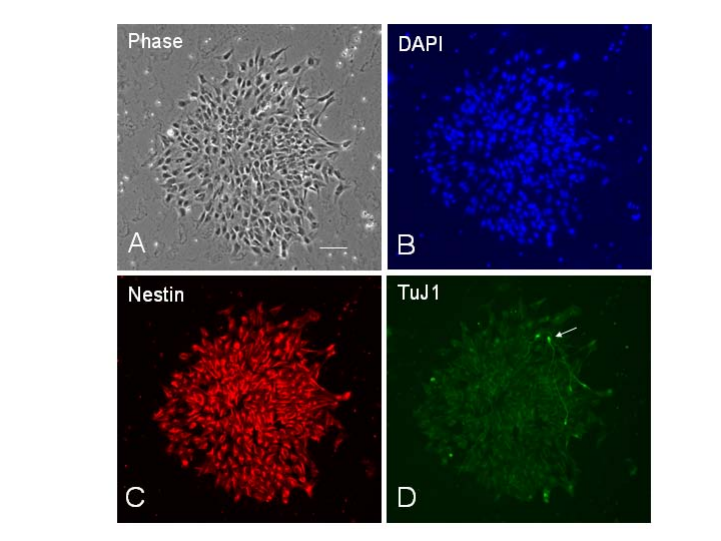
**A homogenous population of nestin+ neural progenitors derived from a light EB**. The light EB derived from TE03 cell line was plated on PDL/laminin and cultured in the NDM for 3 days. To estimate the percentage of differentiated cells expressing nestin or TuJ1, the number of labeled cells was counted from a double-immunolabeled culture at 3 days postplating for nestin (C) and TuJ1 (D), and normalized with the total number of cells determined by counting DAPI stained cells (B) overlapping phase-dark cells (A). This hESC-derived cell population consists of almost all nestin+ progenitors. TuJ1+ neurons (D, pointed by arrows) are barely seen. Scale bar = 100 μm.

Human ES cell lines TE03 and TE06 were maintained and passaged weekly on mitomycin C treated mouse CF-1 embryonic fibroblasts. Colonies of hESCs were removed from feeders, triturated and re-plated in low attachment dishes to obtain spontaneously differentiating EBs. Neuroectodermal differentiation was induced in floating EBs in the neural differentiation medium (NDM). We found a marked change in appearance of differentiating EBs during culturing with the NDM. By 10 days of culture in the NDM (or 15 days of differentiation), most EBs exhibited a solid, dark core surrounded by a light band which we called "dark EBs" (Fig. 1A at 0 hr). However, if continually cultured a few more days in suspension with the NDM, some EBs gradually became transparent capsules which we called "light EBs" (Fig. 1B at 0 hr). Both dark- and light-EBs were plated onto cell culture dishes coated with PDL, PDL/fibronectin, PDL/laminin, collagen and Matrigel. Neural rosettes were readily visualized in plated EBs on all substrates except on PDL. Without clonal isolation of neural rosettes, the neuroectodermal cells in the rosettes further differentiated into neural progenitors, neurons and glia on these adherent substrates. After being plated on PDL/laminin substrates, the dark EBs generated the first nestin+ neural progenitors at 3 hours postplating, and the first TuJ1+ neurons appeared 6 hours after the nestin+ neural progenitors generated. From a dark EB, new neural precursors and neurons were constantly generated and migrated radially away from the center of aggregates, resulting in a rim of cells around the EB sphere (Figs. 1A, 2). While the dark EBs gave rise to a mixed population of nestin+ neural progenitors and TuJ1+ neurons (Figs. 1A, 2), the light EBs produced highly pure nestin+ progenitors with a few or no TuJ1+ cells (Figs. 1B, 3). In both cases, GFAP+ astrocytes and O4+ developing oligodendrocytes did not appear until 4 weeks later. Figure 1 shows time-lapse images of these two distinct differentiation patterns from dark EBs and light EBs respectively.

To quantify the percentage of neural progenitors and neurons within hESC-derived differentiated cells, a double immunocytochemical analysis for nestin and TuJ1 was carried out in cultures with nuclear DAPI counterstaining at 3 days postplating on ECM substrates (or 18 days of differentiation). The dark EBs-derived neural cells exhibited a radial configuration; new neural precursors were constantly generated from the dark EB spheres followed by robust neuronal generation. Both neural progenitors and neurons migrated rapidly away from the center of aggregates (Fig. 2). In the mixed neural population the ratio of nestin+ to TuJ1+ cells varied markedly between EBs-derived aggregates. By counting the cells positive for nestin or TuJ1 and the number of phase-dark cells positive to DAPI in Figure 2, 75% ± 4.2% of differentiated cells expressed nestin and 24% ± 2.1% expressed TuJ1. Figure 3 shows a light EBs-derived neural population. Almost all differentiated cells were nestin+ neural progenitors. By counting the cells positive for nestin or TuJ1 over the number of cells positive to DAPI, 98.2% ± 1.4% of differentiated cells expressed nestin and 1.1% ± 0.4% expressed TuJ1.

### hESC-derived neural rosettes appear on various ECM substrates

**Figure 4:**
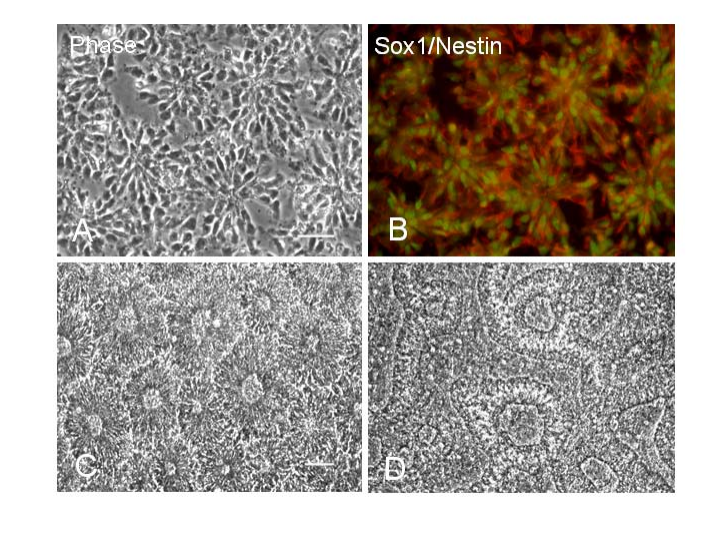
**Human ESC-derived neuroectodermal precursors in neural rosettes (A-B) and neural tube-like structures (C-D)**. The neural rosettes and neural tube-like structures are found in TE-06-derived EBs cultured on PDL/laminin substrates. Neural rosettes are radial arrangements of columnar cells (A) that co-express Sox1 (green) and nestin (red) (B). (C-D) In sister cultures continually fed with NDM for another 3 days, the neural tube-like structures appear. Scale bars: in (A-B) = 50 μm; in (C-D) = 100 μm.

**Figure 5:**
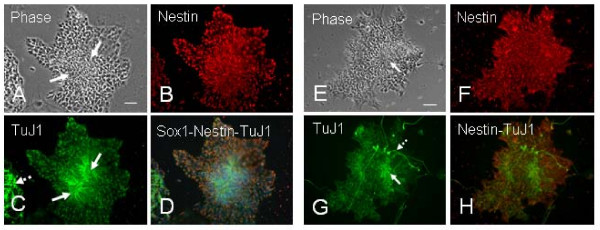
**Neural rosettes generated on collagen (A-D) and matrigel (E-H) substrates**. (A-D) A TE03-derived light EB at 3 days postplating on type I collagen substrates was triple-immunostained for nestin (B), TuJ1 (C) and Sox1 (blue in D). Note that TuJ1 stains radial configurations of structures in rosettes (pointed by two solid arrows) and a cluster of neurons (pointed by a dished arrow) in (C). Scale bar: 100 μm. (E-H) Neural rosettes, neural progenitors and neurons are developed in a TE03-derived light EB cultured on matrigel for 2 days. A solid arrow in (E) points to a TuJ1-labeld radial arrangement (G) which overlaps a rosette in phase contrast image (E). The dashed arrow in (G) indicates TuJ1+ neurons with long processes. The majority of cells in the EB are nestin+ (F). Scale bars in (A) and (E) = 100 μm.

Neuroectodermal precursors in neural rosettes were induced by replating EBs into the NDM. While the markers of undifferentiated hESC such as Oct3/4 and SSEA4 progressively disappeared (not shown), a number of rosettes gradually emerged throughout the EB. Although neural rosettes were seen in floating EBs, more rosettes were generated after the EBs were plated on PDL/laminin (Fig. 4), Matrigel (Fig. 5A–D) and collagen (Fig. 5E–H) substrates, but not on PDL. These radial neuroectodermal cells were morphologically similar to polarized neuroepithelial structures of the developing CNS. Immunostaining of neuroectodermal cells in rosettes for early neural markers Sox1 and nestin, and the neuronal marker TuJ1, showed that the radially organized columnar neuroepithelial cells co-expressed Sox1 and nestin (Fig. 4A, B). Therefore, neural rosettes are considered as *in vitro *organized neuroectoderm [13-15]. It was interesting to notice that the antibody against neuronal marker TuJ1 not only stained rosette cell-derived neurons, but also radially distributed fiber-like structures in rosettes (Fig. 5C, D, G and 5H). About 5–10 days later, the rosettes were often transformed into neural tube like structures (Fig. 4C, D).

### hESC-derived neural progenitors are highly proliferative and multipotent

**Figure 6:**
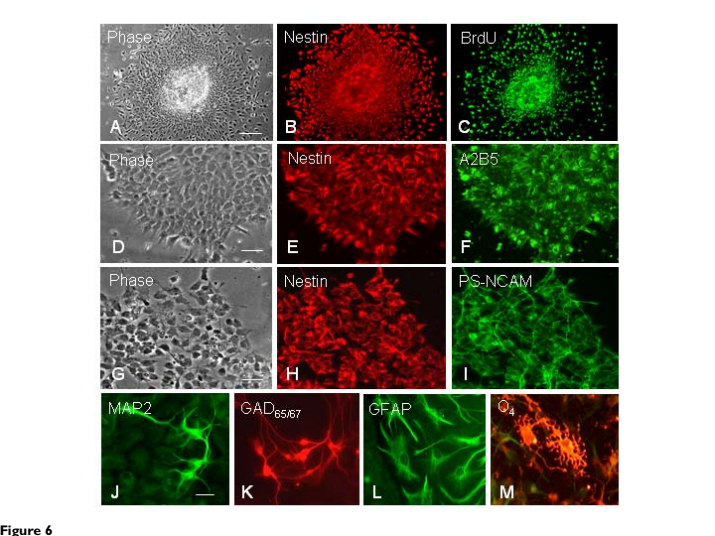
**hESC-derived neural progenitors are highly proliferative and multipotent**. (A-C) Double-immunostaining of differentiated cells at 2 days postplating for nestin (red) and proliferative marker BrdU (greed), indicating approximately 68% of nestin+ cells are BrdU incorporated. (D-F) Double-immunostaining for nestin (red) and A2B5 (green). (G-I) Double-immunostaining for nestin (red) and PS-NCAM (green). The overlapping of nestin and A2B5 or PS-NCAM indicates that hESC-derived nestin+ neural progenitors also express other neural differentiating markers. (J-M) Immunostaining of progeny of hESC-derived neural progenitors for mature neuronal marker MAP2 (J), GABAergic neuronal marker GAD_65/67 _(K), astrocytic marker GFAP (L) and O4, developing oligodentrocyte marker (M). Scale bars: (A) = 200 μm; (D) = 100 μm; (G) = 100 μm; (J-M) = 30 μm.

Our reproducible protocol generated robust hESC-derived neuroectodermal cells giving rise to highly homogenous nestin+ neural progenitors that undergo extensive cell division before differentiating into neuronal and glial cells. To assay the ability of self-renewal of hESC-derived neural progenitors, cells at 18 days of differentiation (or 3 days postplating on laminin substrate) were exposed to BrdU for 4 hours before double immunostaining for nestin and BrdU. The percentage of nuclei positive for BrdU among the nestin-positive cell population was about 68% (Fig. 6A–C), indicating that hESC-derived neural progenitors were actively synthesizing DNA. To characterize the extent to which rosette cells can differentiate into all three neural cell lineages, we used immunocytochemical analysis of cells cultured on PDL/laminin substrates with a panel of antibodies and demonstrated that hESC-derived neural derivatives sequentially expressed Sox1, nestin, musashi (not shown), A2B5 and PS-NCAM, followed by TuJ1 expression in many cells in first 1–5 days postplating on PDL/laminin substrates. MAP2+ and GABAergic (GAD_65,67_+) neurons appeared at about 10–15 days on PDL/laminin substrates. GFAP+ astrocytes and O4+ developing oligodendrocytes did not appear until after 30 days of culture on PDL/laminin substrates (Fig. 6).

### Neural progenitor expansion and differentiation into neurons are significantly greater on laminin or laminin-rich Matrigel

**Figure 7:**
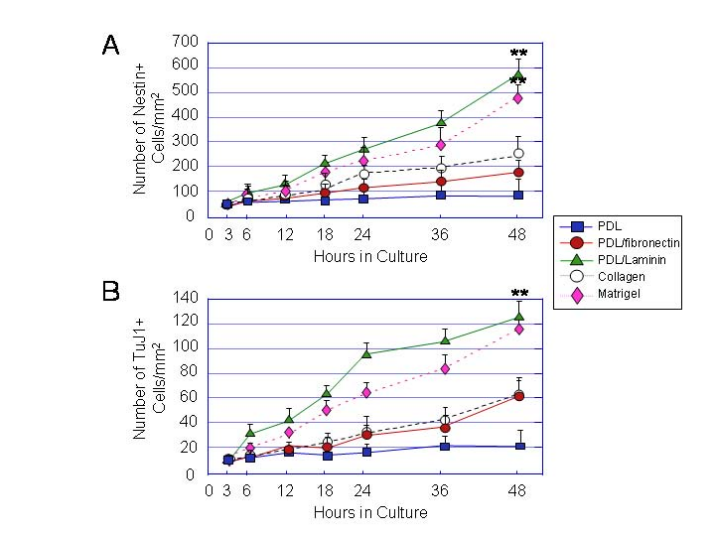
**Expansion of hESC-derived nestin+ neural progenitors and TuJ1+ neurons is greater on PDL/laminin and Matrigel substrates than on PDL, PDL/fibronectin and collagen substrates**. (A) A linear plot summarizing the comparison of neural progenitor expansion on different substrates over time. Values are expressed in the number of nestin+ cells per surface area as mean ± SEM of three independent experiments. The total number of cells per surface area is determined by counting DAPI labeled nuclei which overlapped phase-dark cells. Statistical differences for number of nestin+ cells/mm^2 ^between laminin or Matrigel and PDL or fibrinectin or collagen at 48 h are significant ** p < 0.01. (B) A linear plot summarizing the comparison of neuronal expansion on different substrates over time. Values are expressed in the number of TuJ1+ cells per surface area as mean ± SEM of three independent experiments. The total number of cells per surface area is determined by counting DAPI labeled nuclei which overlapped phase-dark cells. Statistical differences for number TuJ1+ cells/mm^2 ^between laminin or Matrigel and PDL or fibronectin or collagen at 48 h are significant ** p < 0.01.

To examine the effect of substrates on the overall growth (expansion) of neural progenitors derived from dark EBs, similar sized (diameter) EBs with equal number of neural progenitors were chosen at 3 h postplating. The number of nestin+ cells grown on different substrates was quantified over time at 3 h, 6 h, 12 h, 18 h, 24 h, 36 h and 48 h postplating from randomly selected fields. A comparison of cell counts at these 7 time points showed significantly greater cell expansion on PDL/laminin or Matrigel at 12–48 h postplating than on other substrates (Fig. 7A). At 48 h postplating, the nestin+ cell number increased on PDL/laminin by approximately 4-fold and on Matrigel by about 3-fold. A comparison of TuJ1+ cells grown on these 5 substrates also indicated that laminin and Matrigel stimulated significantly higher numbers of hESC-derived neurons compared to the other substrates (Fig. 7B).

### Neurite outgrowth of hESC-derived neurons is greater on laminin and laminin-rich matrigel

**Figure 8:**
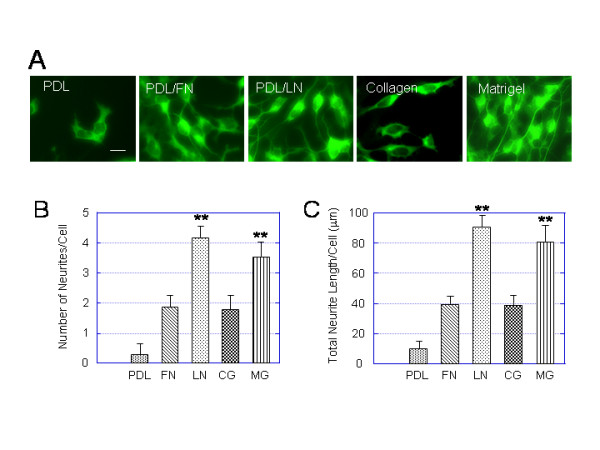
**Neurite outgrowth of hESC-derived neurons are greater on PDL/laminin and matrigel substrates than other substrates**. Light EBs were cultured on 5 different substrates: PDL, PDL/laminin, PDL/fibronectin, collagen and Matrigel for 3 days in the NDM and immunostained for TuJ1. (A) A panel of immunofluorescent images showing representative fields of TuJ1+ cells expanded on PDL, PDL/fibronectin (PDL/FN), PDL/laminin (PDL/LN), collagen and Matrigel for 3 days. (B, C) Bar plots summarizing the effect of different substrates on number of neuritis (B) and total neurite length (C) per cell. The laminin (LN)- and matrigel (MG)-induced number of neuritis and total neurite length per cell are significantly higher than on PDL, fibronectin (FN), and collagen (CG). Values are expressed as mean ± SEM of 4 independent experiments. Statistical differences for number of neuritis and for total neurite length per cell between LM or MG and PDL or FN or CG are significant ** p < 0.01. Scale bar in (A) = 30 μm.

To evaluate the effect of substrates on the neurite outgrowth of hESC-derived neurons, quantification of the number of primary neurites and total neurite length per cell was carried out on neurons derived from the light EBs. The hESC-derived neurons were identified using immunocytochemistry with the antibody against TuJ1 (Fig. 8A). hESC-derived neurons on laminin and Matrigel had significantly greater numbers of neurites and total neurite length per cell than neurons on other substrates (Fig. 8B, C). The total neurite length per cell on laminin slightly greater than on matrigel, but this difference was not statistically significant (total neurite length per cell ± SEM: laminin 85 ± 5.8, Matrigel 81 ± 3.8; P ≥ 0.1 for both comparisons). Therefore, laminin-supported neurite outgrowth was comparable to Matrigel.

### Laminin stimulates neural progenitor expansion and neurite outgrowth in a dose-dependent manner

**Figure 9:**
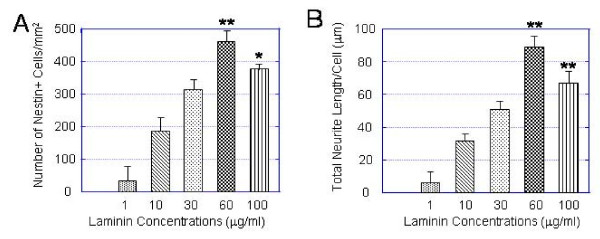
**Laminin stimulates hESC-derived neural progenitor expansion and neuronal neurite outgrowth in a dose-dependent manner**. Dark EB-derived neural populations at 3 days postplating on PDL/laminin substrates with 5 different concentrations: 1 μg/ml, 10 μg/ml, 30 μg/ml, 60 μg/ml and 100 μg/ml. The cultures were double-immunostained for nestin and TuJ1. (A, B) Bar plots summarizing the responses of number of nestin+ neural progenitors (A) and total neurite length of TuJ1+ neurons (B) to different laminin concentrations. The neural progenitor expansion and total neurite length of neurons per cell increases with concentrations and reach a peak at 60 μg/ml. Values are expressed as mean ± SEM of 3 independent experiments. Statistical differences for cells/mm^2 ^and total neurite length per cell between 60 μg/ml or 100 μg/ml and 1 μg/ml or 10 μg/ml or 30 μg/ml are significant * p < 0.05; ** p < 0.01.

Results from above show that laminin is a particularly effective ECM substrate for stimulating hESC-derived neural progenitor expansion and neuronal neurite outgrowth. To examine a possible dose-dependent relationship between laminin concentrations and hESC-derived neurogenesis, we evaluated the hESC-derived neural progenitor expansion and total neurite length of neurons on culture dishes coated with PDL/laminin substrates. Laminin was used at the following 5 concentrations: 1 μg/ml (0.25 μg/cm^2^), 10 μg/ml (2.5 μg/cm^2^), 30 μg/ml (7.5 μg/cm^2^), 60 μg/ml (15 μg/cm^2^) and 100 μg/ml (25 μg/cm^2^). We observed that both neural progenitor expansion and total neurite length increased in response to increased laminin concentrations from1 μg/ml to 60 μg/ml. The cell expansion and neurite outgrowth declined at higher laminin concentration (100 μg/ml) (Fig. 9A, B).

### Distributions of integrins α1, α3, α6, β1 and β4 in hESC-derived neural progenitors

Integrins comprise a large family of cell adhesion molecules that mediate interactions between the extracellular environment and the cytoplasm. Since hESC-derived neural progenitors were predominantly interacting with laminin, we examined if the neural progenitors express the laminin-associated integrin subunits. It is known that integrin β1 is widely expressed in human postnatal cortices-derived neural progenitor cells [16]. It forms noncovalent complexes with various integrin alpha subunits, including α1, α3, α6, to become the functional receptors that bind specifically to laminin. Flow cytometry showed that the human neural progenitor cells express the laminin-associated integrins α1, α3, α6, β1 and β4 [16]. We carried out immunostaining of hESC-derived neural progenitors at 3 days postplating on PDL/laminin for integrins α1, α3, α6, β1 and β4. All these integrin subunits were detectable to differing degrees. Cell counting of immunoreacted vs total number of cells revealed that 98% ± 1.5% cells expressed β1 subunit and 46% ± 8% cells expressed α6 subunit. There were 38% ± 4% α3 positive cells, 12% ± 3% α1 positive cells, and 15% ± 3% β4 positive cells found in hESC-derived neural progenitors.

### Blockage of α6 or β1 integrin disrupts expansion of hESC-derived neural progenitors on laminin substrates

**Figure 10:**
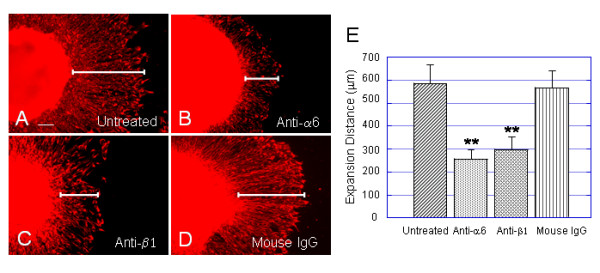
**Anti-α6 or β1 integrin antibody blocks laminin-induced neural progenitor expansion**. Antibody perturbation experiments were carried out in dark EB-derived neural populations at 24 h postplating on laminin substrate at 60 μg/ml. A 10 μg/ml of antibody against α6 or β1 integrin, and a 10 μg/ml of purified mouse IgG were added into the culture medium directly at the beginning of cell culture. The expansion distance was determined by measuring the length from the edge of EB spheres to the widest point of the rim. (A-D) Immunofluorescent images of dark EB-derived neural populations show representative fields of nestin+ cells treated with anti-α6 (B), anti-β1 (C), mouse IgG (D), and untreated (A). Inclusion of antibody against α6 or β1 significantly decreases cell expansion compared to untreated culture (A) and culture treated with mouse IgG (D). The bar in (A) = 503 μm, in (B) = 231 μm, in (C) = 266 μm, and in (D) = 466 μm. (E) Bar plot summarizes the effects of various treatments on laminin-induced expansion distances. Values are expressed in expansion distance as mean ± SEM of four independent experiments. Statistical differences for expansion distance between anti-α6 or β1 integrin treatment and untreated or treated with mouse IgG are significant: **p < 0.001. But the differences for expansion distance between mouse IgG treatment and untreated are not significant p > 0.05. Scale bar in (A) = 100 μm.

Results above demonstrated that, compared with other substrates, hESC-derived neural progenitors showed strongest responses to laminin substrates in their migration, expansion and differentiation into neurons. Recent studies have shown that almost all human neural stem and progenitor cells express the β1integrin subunit and a significant percentage of human neural progenitors express a6 subunit [16]. To assess the role of endogenous a6 and β1integrins in hESC-derived neural progenitor migration and expansion on PDL/laminin substrates, antibody perturbation experiments were performed in dark EBs-derived neural populations cultured at 24 h postplating. We marked the similar sized (diameter) dark EBs at the day of plating (or at 15 days after differentiation) in total 12 cell culture dishes coated with PDL/laminin at concentration of 60 μg/ml. These dishes were divided into 4 groups: no treatment (Fig. 10A), treated with the antibody against a6 integrin (Fig. 10B), treated with the antibody against β1 integrin (Fig. 10C) and treated with purified mouse IgG (Fig. 10D). Cell expansions were estimated at 24 h postplating by measuring expansion distances from the edge of the EB sphere to the widest point of the rim (Fig. 10A–D). It was found that hESC-derived neural progenitors treated with a6 or β1integrin antibody exhibited significant decreases in cell expansion distances (expansion distance ± SEM: a6 treated 256 ± 26 μm; β1 treated 301 ± 32 μm) compared to non-treated cells (589 ± 71 μm) or cells treated with mouse IgG (574 ± 65 μm). Cells untreated or treated with mouse IgG showed no significant difference in cell expansion, whereas statistical differences between a6 or β1 integrin antibody-treated cells and values for control cultures were found significant (** p < 0.01). This result indicates that a6β1integrin mediated, at least in part, hESC-derived neural progenitor responses to laminin, suggesting a critical role for laminin/a6 or β1integrin signaling in hESC-derived neural progenitor cell migration and expansion.

## Discussion

Our work demonstrates that laminin and laminin-rich Matrigel significantly enhanced directed hESC differentiation into neural progenitors and neurons compared with PDL, fibronectin and type I collagen. Robust neuroectodermal precursors in neural rosettes were formed on laminin substrates and differentiated into greater numbers of neural progenitors and neurons with greater neurite outgrowth. We also found that laminin stimulated neural progenitor expansion and neurite outgrowth in a dose-dependent manner; the antibody against α6 or β1integrin subunit partially blocked the laminin-stimulated expansion of hESC-derived neural progenitors. These results indicate that the endogenous α6 and β1 integrin subunits were in part responsible for transmitting laminin signaling into the cell to stimulate hESC-derived neural progenitor expansion and neuronal differentiation, suggesting that laminin/α6β1integrin signaling plays a significant role in the directed neural differentiation of hESCs.

The directed neural differentiation of hESCs in culture is a dynamic process which is essentially a recapitulation of early embryonic developmental processes [17]. Our results from neural differentiation of hESCs *in vitro *are consistent with recent findings from *in vivo *studies. Evidence has shown that embryonic neural tissues contain a dynamic ECM, composed of many types of molecules that have distinct patterns of spatial and temporal expression. The laminin family is one of the most important ECM components within the neural stem cell niche. Neural stem cells derived from the mouse VZ, SVZ and the rostral migratory stream (RMS) to the olfactory bulb express high levels of integrin α6β1 and its ligand laminin [10,18]. Antibodies against the α6 or β1 integrin subunits can disrupt neuroblast migration, suggesting an endogenous role for α6β1 integrin in guiding migration; laminin is a chemoattractant for neuroblasts of the SVZ/RMS, drawing neuroblasts away from their normal course of migration in a restricted fashion when injected as a tract of intact laminin, and in a dispersed fashion when provided as a more soluble peptide [18]. The latter studies emphasize a critical role of α6β1 integrin and its ligand, laminin, in controlling the direction of migrating neuroblasts in the adult CNS.

The responses of hESC-derived neural progenitors to ECM molecules found in the present study are similar to those of neural stem/precursor cells dissociated from human postnatal cortices [16] or from mouse ganglionic eminence [7]. In the latter studies, laminin matrices enhanced human neural stem cell migration, expansion and differentiation into neurons and astrocytes, and elongation of neurites from NSPC-derived neurons compared to poly-L-ornithine and fibronectin. Ours and other studies indicate laminin interactions with α6β1 integrin represent one of the most important cell-ECM interactions as an early inductive signal to regulate neural stem and progenitor cell proliferation, migration and fate decision *in vitro *and within the embryonic mammalian brain.

In the present study we focused on the involvement of the ECM components in the generation and migration of neural progenitors and neurite outgrowth of neurons during the neural specification of hESC. Laminin served as a potent stimulator of neural differentiation of hESCs while other tested ECM components showed similar but much less enhancement of these events. Recent evidence has increased our awareness of the diversity and complexity of the cell-matrix interactions. ECM components have both stimulatory and inhibitory effects on neural cell development [19], and much remains to be elucidated about how interactions with the ECM generate inductive signals that regulate the hESC differentiation *in vivo *and *in vitro*. On the other hand, cells in tissues encounter the composition of the ECM, rather than a single ECM protein. For example, the basement membrane and connective tissue contain networks of multiple ECM proteins including laminin, fibronectin and several collagen isoforms. Study of the role of combined ECM components, which regulate hESC-derived neural phenotypes, will greatly facilitate their use as model systems for neural development study and as therapeutic agents for cell replacement.

## Conclusion

We demonstrated using a defined adherent culture system that neural derivation from hESCs was significantly enhanced by laminin or laminin-rich Matrigel compared to PDL, fibronectin and type I collagen substrates. Laminin stimulated neural progenitor generation, expansion and differentiation into neurons, as well as neurite outgrowth of hESC-derived neurons. The laminin-induced neural progenitor expansion was partially blocked by the antibody against α6 or β1 integrin. These results implicate a possible role of laminin/α6β1 integrin signaling in directed neural differentiation of hESCs. This finding is consistent with a significant role of laminin/integrin signaling in regulation of neural stem cell generation, migration and differentiation within the ventricular zone of CNS. Thus, cell-ECM interactions appear to be an early inductive signal to regulate neural specification of hESCs. Our findings may facilitate studies of early human CNS development and potential applications in neurological diseases.

## Methods

### *In vitro *maintenance and propagation of undifferentiated TE03 and TE06 hESCs

Human ESC lines TE03 (XX, passages P29 – P35) and TE06 (XY, approximately passage 35) were cultured according to the guidelines established by the National Academy of Sciences. Cells were propagated on a feeder layer of Mitomycin C. treated Mouse embryonic fibroblasts CF-1 (ATCC SCRC-1040.2; Manassas, VA; ). Cells were cultured at 37°C, 5% CO_2 _in ES medium-Dulbecco's modified Eagle's medium (D-MEM)/F12 (ATCC 30-2006) (80%), supplemented with 2.0 mM L-Alanyl-L-Glutamine (ATCC 30-2115), 0.1 mM Non-essential amino acids (ATCC 30-2116), 0.1 mM 2-mercaptoethanol (Sigma Catalog No. M-7522; ) and 4 ng/ml basic fibroblast growth factor (bFGF) (R& D Systems Catalog No. 233-FB) (5%); Knockout serum replacement (Invitrogen Catalog No. 10828) (15%); fetal bovine serum (ATCC SCRR-30-2020), Penicillin (100 IU/ml)/streptomycin (100 μg/mL) (ATCC 30-2300). bFGF (4 ng/ml) was added in the first 24 hours after thawing the cells. Daily medium changes began after the first 48 hours in culture. Cells were passaged every 6 to 8 days using collagenase IV (200 Units/ml) (Invitrogen, Carlsbad, CA; ).

It has been a standard practice in our laboratory to monitor these cell lines by repeated sterility testing that ensures the cell cultures were free from mycoplasma, bacteria, fungi and viral pathogens.

### Spontaneous and directed neural differentiation and rosette formation

The hES cell colonies were removed from MEF feeders and dissociated into small clumps by incubating with collagenase IV (200 Units/ml) (Invitrogen Carlsbad, CA; ) at 37°C for 30 minutes. The hES cell clumps were pelleted and cultured in suspension in low attachment dishes with hES cell medium without bFGF for 5 days (the end of this stage is considered as at 5 days of differentiation). ES cells grew into floating aggregates or embryoid bodies (EBs), while remaining feeder cells adhered to the plate. The neuroectodermal induction began with EBs transferred into the neural differentiation medium (NDM) that consisted of modified Eagle's medium (ATCC 30-2002; ), F12k-(ATCC 30-2004), N-2 supplement (Gibco Catalog No. 317740; ), 0.1 mM Non-essential amino acids (ATCC 30-2116), Penicillin (100 IU/ml)/streptomycin (100 μg/ml) (ATCC 30-2300) and 5 ng/ml bFGF (R& D Systems Catalog No. 233-FB) for 10 days. At days 15–17 of differentiation, EBs were plated on substrate-coated 35 mm dishes. Although some neural rosettes were formed in floating EBs, increased rosettes were visualized after plating of the EBs on substrates. Neuroectodermal cells in neural rosettes further differentiated into neural progenitors and their progeny. Immunocytochemistry of neuroectodermal cells, neural progenitors and their progeny was carried out directly on substrates.

### Substratum preparations

To examine ECM effects on neural differentiation of hESC *in vitro*, the treated polystyrene 35 mm cell culture dishes (Corning, NY; ) were coated with the following substrates: Poly-D-Lysine (PDL), human placenta-derived fibronectin (FN), human placenta-derived laminin (LN), type I collagen and Matrigel. PDL, a positively-charged, synthetic molecule, was used to enhance cell attachment to plastic surfaces and enhance adsorption of ECM proteins to the culture substrate. In addition, we used human ESC Qualified Matrigel Matrix (BD Biosciences, Bedford, MA; ), a solubulized basement membrane preparation extracted from EHS mouse sarcoma (a tumor rich in ECM proteins); its major component is laminin (56%), followed by collagen IV (31%), heparan sulfate proteoglycans, and entactin (8%). It provides a physiologically relevant environment for stimulation of cell proliferation and differentiation. Matrigel has been useful for studies of cell morphology, biochemical function, migration or invasion, and gene expression.

PDL (Sigma-Aldrich, St. Louis, MO; ) was reconstituted with sterile ddH_2_0. The PDL solution was added to plates at 0.1–1 mg/ml and left overnight at room temperature. For laminin and fibronectin coating, dishes were pretreated with PDL as described above, fibronectin derived from human plasma (R & D Systems, Minneapolis, MN; ) was added at 50 μg/ml in PBS, and laminin from human placenta (Sigma-Aldrich, St. Louis, MO; ) as added at 10–100 μg/ml in PBS. PDL, PDL/fibronectin, and PDL/laminin were left on dishes overnight in a humidified 37°C, 5% CO2 incubator, and then the excess substrate was removed and dishes were rinsed with PBS.

To examine the effects of laminin concentrations on hESC-derived neurogenesis, we systematically measured neural progenitor expansion and total neurite length per neuron at 5 laminin coating concentrations: 1 μg/ml (0.25 μg/cm^2^), 10 μg/ml (2.5 μg/cm^2^), 30 μg/ml (7.5 μg/cm^2^), 60 μg/ml (15 μg/cm^2^) and 100 μg/ml (25 μg/cm^2^).

Collagen (Type I, Boehringer-Mannheim Corp., Indianapolis, IN; ) purchased as a sterile, lyophilized powder, was dissolved to a final concentration of 3 mg/ml by addition of sterile 0.2% v/v acetic acid (pH 3–4). Then the collagen solution was diluted with an equal volume of 2× phosphate-buffered saline (PBS, Gibco; ) and a volume of cell media to achieve a final collagen concentration of 0.5 mg/ml (maintaining physiological osmolarity, 250–300 mOsM). After adjusting the pH of the collagen solution to pH 7.4 by the addition of 1 N NaOH, the solution was chilled in an ice bath to prevent gel formation. The collagen solution can be simply applied to 35 mm dishes or glass coverslips and allowed to dry.

Human ESC Qualified Matrigel Matrix was thawed overnight at 4°C then diluted 1:6 (approximately 16% Matrigel) in cold DMEM/F12 medium (ATCC, Manassas, VA; ). 35 mm cell culture dishes were coated on ice so as not to gel the Matrigel prematurely. The volumes of 16% Matrigel used to coat the tissue culture vessels were 1 ml per 35 mm dish and 0.25 ml per well in the 24 well plates. Culture dishes were kept on ice for 1 hour, and excess Matrigel was aspirated just prior to plating of neural progenitor cells.

### Immunocytochemistry

To evaluate the neural differentiation potential of pluripotent hESCs, immunostaining for various neural markers was performed. The staining procedure was described previously [20]. Briefly, cultured cells were fixed for 15 min in 4% paraformaldehyde in PBS followed by incubation with the primary antibodies at 4°C overnight. Appropriate secondary antibodies were added for single or double staining.

The following primary antibodies were used: rabbit anti-nestin (Millipore/Chemicon, Billerica, MA; ; 1:200), mouse Class IgG_2a _anti-β-tubulin (TuJ1) (Millipore/Chemicon, Billerica, MA; ; 1:300), chicken polyclonal anti-Sox1 (Millipore/Chemicon, Billerica, MA; ; 1:300), rabbit polyclonal anti-musashi (Millipore/Chemicon, Billerica, MA ; 1:300), mouse Class IgM anti-A2B5 (R&D Systems, Minneapolis, MN; ; 1:100), mouse Class IgM anti-PSA-NCAM (Millipore/Chemicon, Billerica, MA; ; 1:300), mouse Class IgG1 anti-MAP2 a, b, c (Kamiya Biomedical company, Seattle, WA; ; 1:100), rabbit glutamate decardoxylase (GAD)_65/67 _(Millipore/Chemicon, Billerica, MA, ; 1:200), mouse Class IgM clone 81 anti-O4 (Millipore/Chemicon, Billerica, MA; ; 1:100) and rabbit polyclonal anti-GFAP (Millipore/Chemicon, Billerica, MA ; 1:500). Secondary antibodies were FITC-conjugated donkey anti-mouse IgG_2a _(Southern Biotech, Birmingham, AL; ; 1:50), rhodamine-conjugated donkey anti-rabbit IgG (Jackson ImmunoResearch, West Grove, PA; ; 1:50), FITC-conjugated donkey anti-chicken IgG (Millipore/Chemicon, Billerica, MA, ; 1:50), FITC-conjugated donkey anti-Mouse IgM (Jackson ImmunoResearch, West Grove, PA; ; 1:50) or AlexaFluor488 conjugated goat anti mouse IgG1 (Invitrogen/Molecular Probes, Eugene, OR; ; 1:50). Cell nuclei were labeled for 10 min with 0.8 μg/ml 4,6-diamidino-2-phenylindole (DAPI) (Sigma-Aldrich, St. Louis, MO; ).

To examine the distributions of integrins in hESC-derived neural progenitors, immunocytochemistry for laminin-associated integrins α1, α3, α6, β1 and β4 was performed in neural progenitors generated on PDL/laminin substrates. Antibodies against integrins α1 (clone MB1.2), α3 (clone ASC-6), α6 (clone NK1-GoH3), β1 (clone HUTS-4) and β4 (clone 3E1) were purchased from Chemicon (Millipore/Chemicon, Billerica, MA; ; 1:100). Secondary antibody was FITC-conjugated donkey anti-Mouse IgG (Jackson ImmunoResearch, West Grove, PA; ; 1:50). The distributions of immunofluorescent cells were examined under microscope (Nikon) eclipse TE 300 microscope with Bioimaging System.

### Double-immunocytochemistry for nestin and BrdU incorporation

To examine the ability of hESC-derived neural progenitors (nestin+ cells) to synthesize DNA, bromodeoxyuridine (BrdU) incorporation with 5-bromo-2-deoxy-uridine labeling and Detection Kit I (Roche, Indianapolis, IN; ) was used to monitor nestin+ cell proliferation. Cultures were exposed to 20 μM BrdU for 4 hours and then fixed with 70% alcohol containing 50 mM glycine at PH 2.0. After rinse, cells were incubated overnight with mouse anti-BrdU (1:1000) and rabbit anti-nestin (Millipore/Chemicon, Billerica, MA; ; 1:200), followed by incubation with a mixture of rhodamine-conjugated donkey anti-rabbit IgG and FITC-conjugated donkey anti-mouse IgG (Jackson Immunological Research, West Grove, PA) for 45 min. Some cultures not exposed to BrdU were used as negative controls and showed no immunoreactivity demonstrating the specificity of BrdU antibody. In the double-labeling experiment, no cross-reactivity was detected between BrdU and anti-nestin antibodies. The distributions of double-immunostained nestin+ and BrdU+ cells were examined and photographed with Nikon eclipse TE 300 microscope. A proliferation index was defined as the percentage of BrdU+ nuclei in the total number of cells evaluated. At least 500 labeled cells were counted from each dish.

### Antibody perturbation experiments

To test the role of α6 or β1 integrin in laminin-stimulated hESC-derived neural progenitor cell expansion, hESC-derived dark EBs were plated on PDL/laminin substrates and cultured in the NDM. A 10 μg/ml of antibodies against α6 or β1 integrin (Anti-Integrin alpha6, clone NKI-GoH3 from Chemicon; ; or Affinity purified anti-mouse integrin β_1 _from eBioscience ) and a 10 μg/ml of purified mouse IgG (Chemicon) were added into the culture medium directly at the beginning of cell culture on a PDL/laminin substrate. Anti-integrin α6, clone NKI-GoH3 is well characterized antibody which has been used to inhibit laminin binding [21,22]. Affinity purified anti-mouse integrin β_1 _clone HMb1-1 has been reported to block VLA-dependent cellular functions, including the adhesion of mouse tumor cell lines to extracellular matrix proteins [23,24]. Compared to monoclonal anti-human integrin β1/CD29 antibody clone P5D2 (R&D systems, ) and anti-β1 integrin antibody clone TASC/9D11, (Chemicon, ), affinity purified anti-mouse integrin β_1_exhibited greater blocking effects, and was used for this study. Measurements of cell expansion were carried out in similar sized (diameter)-dark EB-derived neural populations at 24 h postplating.

### Quantification of hESC-derived neural cells and neurite outgrowth

To quantify the percentage of hESC-differentiated neural progenitors and neurons, cell counting was performed from cultures double-immunostained for nestin and TuJ1, together with nuclear DAPI counterstaining, in 35 mm culture dishes coated with different ECM substrates from at least three independent experiments. Nestin+ and TuJ1+ cells were manually counted and were expressed as a percentage of the total differentiated cells. We found that DAPI stains both differentiated and undifferentiated cells, but differentiated cells usually exhibit phase-dark under microscope. Therefore, only those DAPI stained cells overlapped with phase-dark cells were counted as total differentiated cells. At least 2,000 total cells were evaluated per dish for one antigen expression. Two methods were used for cell expansion measurements. First, we counted the number of immunostained cells on different substrates from randomly selected fields. Second, we measured expansion distances in dark EBs-derived differentiation population in which the distance from the edge of the EB sphere to the widest point of the rim can be measured.

Neurite outgrowth was analyzed using the NIH image analysis software. Neurites were quantifies from images of neurons immunostained for TuJ1. Measurements of neurite outgrowth included the number of primary neurites per cell and total neurite length per cell. A neurite was defined as a process having longer than the width of one cell body terminating in a growth cone and neurites were recorded using trace function. The total number of neuritis and total neurite length were measured and divided by the number of cells observed in the field. The total neurite length per cell was calculated by averaging the sums of analysis. For these analyses, each captured image was identified as a sampling unit and data from three separate experiments were pooled. An average of 25 cells per image was analyzed in each experiment.

All data are expressed as mean ± SEM, and Student's *t *test was used for statistical evaluation. In all instances * P < 0.05 was considered statistically significant.

## Authors' contributions

WM, MR and MM were primarily responsible for the data analysis and writing and editing the manuscript. TT, ED and JS carried out the hES cell cultures and directed differentiation, and performed immunocytochemistry.
